# Injection of the Broad Ligaments for Prolapsus Uteri

**Published:** 1911-07-29

**Authors:** J. Inglis Parsons

**Affiliations:** Surgeon to the Chelsea Hospital for Women.


### Hospital Clinics.

### INJECTION OF THE BROAD LIGAMENTS FOR PROLAPSUS UTERI.

By J. INGLIS PARSONS, M.D., M.R.C.P., M.R.C.S., Surgeon to the Chelsea Hospital for Women.

The idea of this operation is to induce repair in the ligaments of the uterus by stimulation. The movements of the uterus and its adaptation to the movements of the bladder and intestines, without any consciousness of friction in the healthy subject, resemble very closely the movements of the opposing surfaces in an ordinary joint. When dislocation occurs in the latter it is nearly always produced suddenly as the result of violence. Such sudden violence and injury to the cells form the stimulus which produces the inevitable swelling and subsequent repair. On the other hand, the uterus is very rarely suddenly dislocated. The ligaments are gradually stretched, and the process is spread over many months, or even years, so that there is never at any particular time sufficient injury to act as a stimulus to repair. In confirmation of this it may be pointed out that gynæcologists operating on the uterus for conditions other than prolapse very often forcibly drag the cervix down to the vaginal outlet. Prolapse never results from this, because the sudden dislocation acts as a stimulus to repair.

### The Anatomy of the Uterus.

Until within recent years it was thought that the pelvic floor keeps up the uterus. Twenty-nine years ago the late Dr. Henry Savage, of the Samaritan Hospital, in his book, "On the Anatomy of the Female Pelvic Organs," pointed out that after division of the utero-sacral ligaments and round ligaments, the uterus is still held up by the sub-peritoneal cellular tissue, particularly where it surrounds and accompanies the uterine blood vessels. His work and experiments fell on deaf ears. It was not until a German anatomist brought forward the same fact fourteen years later that notice was taken of it. Even recently the writer of a paper read at the Royal Society of Medicine on the supports of the uterus was quite unaware of Dr. Savage's work.

Another anatomist whose work has been of great service to me is Dr. Clarence Webster. In his book on "Researches in Female Pelvic Anatomy," published in 1892, p. 87, he laid great stress on the importance of the connective tissue attaching the cervix to the side walls of the pelvis, and also to the muscular and elastic tissue in the same position. It is this utero-pelvic band running from the side of the uterus to the obturator fascia that I make use of in my operation to sustain the uterus. Dr. Savage also pointed out that the round ligaments have but little influence in holding up the uterus. This was proved by cutting away all the other supports and then trying how far the round ligaments allowed the uterus to come down.

Since retroversion is often associated with prolapse, it is important to have a clear idea not only of the supports of the uterus against prolapse, but also against retroversion. This latter displacement is prevented by the round ligaments, while Savage's ligaments prevent prolapse. When prolapse occurs without retroversion it is only Savage's ligaments that have given way. These are the best cases for injection with quinine.

### Retroversion.

When retroversion occurs alone without prolapse it is due to the giving way of the round ligaments, while Savage's ligaments hold up the uterus. In other words, the round ligaments prevent retroversion but do not prevent prolapse. Savage's ligaments prevent prolapse but do not prevent retroversion. All these statements can be demonstrated on the cadaver by cutting away one set of ligaments at a time. When the prolapse is associated with retroversion, all the ligaments have given way. To meet the latter class of case it is necessary for the patient to wear a Hodge pessary for a time after the prolapse is cured, and in most instances in the course of six to twelve months the retroversion disappears.

It is further necessary to appreciate the fact that the strength of the ligaments varies very widely in different women. At one end of the scale is a young patient of mine, age twenty-one, and single, with the cervix trying to protrude through an intact hymen. At the other end, a woman of forty, who has had a dozen children, and also a complete tear of the perineum going well into the rectum for fourteen years, and yet the uterus has not shown the slightest tendency to prolapse or retrovert. Between these two extremes are an infinite number of gradations. A not uncommon type is the woman who is on the borderland of prolapse. As long as her parturitions are quite normal and her general health good, with no adverse conditions of life, she escapes prolapsus. But if her general health is run down after childbirth, by doing too much, getting up too soon, an attack of influenza, or a torn perineum, the ligaments then begin to give way.

### Perineal Tears.

The reason why a torn perineum is a factor in these cases is because the vaginal walls begin to protrude from want of their normal support, namely, the perineum, and as the protrusion goes on they drag on the uterus. If the ligaments of the latter under these conditions have no margin of strength to spare, they begin to give way and prolapse commences. When, under these conditions, the perineum is promptly restored, the drag is taken off the uterus and the ligaments *may* recover themselves. It was this class of case and the result that followed from restoring the perineum that gave rise to the erroneous view that the pelvic floor kept up the uterus.

In order to do something for the large number of cases that were not cured by perineorraphy the operation of ventrifixation was introduced, but there is more than one objection to it.

In order to hold the uterus up, if the prolapse is beyond the first stage, it is necessary really to fix it with several sutures to the abdominal wall. The result of this is that in a large percentage of cases when the woman becomes pregnant the uterus has a difficulty in expanding, and abortion occurs. If the uterus manages to expand, there may still be considerable distortion at the time of parturition, which gives rise to difficulty in delivering the child.

After the menopause ventrifixation is in most cases out of the question, because the abdominal wall is not strong enough to take the strain. Very often the uterus fails to adhere, or if it does adhere it drags down the abdominal wall into a pouch, which is almost as uncomfortable to the patient as the procident uterus.

For minor cases of prolapse associated with retroflexion, ventro suspension, not fixation, is quite satisfactory. Only two silk sutures are used. When these are absorbed, a thin adhesion forming a suspensory ligament about an inch to an inch and a half in length is formed. This keeps the uterus in place and does not prevent expansion during pregnancy, and, so far as my own experience of twenty years has gone, no other trouble has been caused by it.

Vaginal fixation, which has had a fair trial by now, has been to a great extent abandoned on account of the difficulties it produced in subsequent parturition, and because in many cases the patient's condition was not much better than before.

To do hysterectomy is a confession of failure, but it may be necessary occasionally.

None of these objections can be brought against injection of the broad ligaments. The anatomy of the pelvis is not in any way interfered with. There is no band in the peritoneal cavity to cause intestinal obstruction. There is no drag on the abdominal wall. There is no interference with the expansion of the uterus or bladder. There is no obstruction to parturition. The operation will even succeed in some cases of chronic procidentia when the only other alternative is hysterectomy. But as the basis of my operation is physiological, it requires a greater amount of clinical acumen than is necessary for a mere mechanical operation like vaginal or abdominal fixation. The injection of quinine, or some other irritant, is made with a fine needle into the utero-pelvic ligament through the vaginal wall, and is fully described in my book. This produces an effusion of lymph, which in course of time becomes organised into fibrous tissue and holds up the uterus. The process is, in fact, the same as that which takes place when the ligaments are repaired in an ordinary joint after dislocation, and also *takes as much time* before repair is complete.

To obtain success, some opinion must be formed of the capacity of the patient to respond to a stimulus to repair. This I have found to vary through wide limits. Speaking broadly, a good constitution and a vigorous habit of body and a country life produce better results than the opposite conditions.

When we have to deal with a woman of bad family history, who is perhaps also anæmic or worn out by frequent child-bearing, we may be fairly sure that in such condition she will not respond to the injection. Before advising operation, the anæmia must be treated by a course of iron, a plentiful meat diet with some burgundy or port wine, and, if possible, a visit to the country for some weeks will be found to improve matters considerably.

The more difficult questions to decide are the estimation of the amount of effusion and whether it is sufficient for the purpose, and also to know how soon the patient may be allowed to get up without risk of over-stretching the new ligaments before they have become strong enough to take the strain.

Here again several factors have to be considered. A small amount of effusion of lymph after the injection may be quite sufficient to hold up the uterus permanently in an early case, but not sufficient in a chronic case.

I am often asked how long must patients lie up after the operation, as if all cases were alike. It depends on the duration and extent of the prolapse, and also on the reaction of the patient to the injection.

For an early case of prolapse ten days is usually sufficient, and even that time need not all be spent in bed. Consider the difference between the condition of the ligaments in an early case just beginning to stretch and that of a case of chronic procidentia, with the ligaments elongated to double their usual length, the vaginal walls relaxed, stretched, and prolapsed, the uterus enlarged, eroded, and much heavier than normal, the bladder and the rectum dragged out of place, with their particular supports also gone. It stands to reason that a much larger effusion of lymph and a much longer stay in bed is necessary in a case of this kind than in the former.

The after-success of the operation will depend in a great measure on the intelligent appreciation of these factors. Success may often be obtained in feeble patients who do not react well by keeping them at rest for a longer time and also by making them wear an efficient pessary for some months so as to take the strain of the uterus off the ligaments, until the small effusion has had time to become converted into strong fibrous tissue. When the perineum is ruptured and the vaginal walls are prolapsed they drag on the uterus and tend to pull it down again, so the cure is materially assisted by sewing up the perineum. Although the perineum in no way affords any direct support to the uterus, it most certainly does to the vaginal walls, and this prevents them dragging on the uterus and its ligaments.

(*To be continued.*)

